# Influence of preprocedural glycemic control on clinical outcomes of endovascular therapy in diabetic patients with lower extremity artery disease: an analysis from a Korean multicenter retrospective registry cohort

**DOI:** 10.1186/s12933-020-01072-x

**Published:** 2020-06-22

**Authors:** Jung-Joon Cha, Hyoeun Kim, Young-Guk Ko, Donghoon Choi, Jae-Hwan Lee, Chang-Hwan Yoon, In-Ho Chae, Cheol Woong Yu, Seung Whan Lee, Sang-Rok Lee, Seung Hyuk Choi, Yoon Seok Koh, Pil-Ki Min, K-VIS (Korean Vascular Intervention Society) investigators, Woong Chol Kang, Sung-Ho Her, Yoon Seok Koh, Byung-Hee Hwang, Byung-Hee Hwang, Ae-Young Her, Weon Kim, Cheol Woong Yu, Sang Cheol Jo, Sang Cheol Jo, Sanghoon Shin, Yun Hyeong Cho, Woo-Young Chung, In-Ho Chae, Chang-Hwan Yoon, Jung Kyu Han, Seung Whan Lee, Seung Hyuk Choi, Young Jin Choi, Su Hyun Kim, Sang Ho Park, Pil-Ki Min, Donghoon Choi, Young-Guk Ko, Young Jin Yoon, Jung-Hee Lee, Yu Jeong Choi, Sung Kee Ryu, Ju Han Kim, Sang-Rok Lee, Hoyoun Won, Ju Yeol Baek, Jae-Hwan Lee, Jang-Hwan Bae, Hyun-Sook Kim

**Affiliations:** 1 Division of Cardiology, Department of Internal Medicine, Severance Cardiovascular Hospital, Yonsei University College of Medicine, Seoul, South Korea; 2 Division of Cardiology, Department of Internal Medicine, Chungnam National University Hospital, Daejeon, South Korea; 3 Division of Cardiology, Seoul National University Bundang Hospital, Seoungnam, South Korea; 4 Present Address: Division of Cardiology, Department of Internal Medicine, Korea University Anam Hospital, Seoul, South Korea; 5 Division of Cardiology, Department of Internal Medicine, Asan Medical Center, University of Ulsan College of Medicine, Seoul, South Korea; 6 Division of Cardiology, Department of Internal Medicine, Chonbuk National University Hospital, Jeonju, South Korea; 7 Division of Cardiology, Department of Medicine, Samsung Medical Center, Sungkyunkwan University School of Medicine, Seoul, South Korea; 8 Division of Cardiology, Department of Internal Medicine, Seoul St. Mary’s Hospital, College of Medicine, The Catholic University of Korea, Seoul, South Korea; 9 Division of Cardiology, Department of Internal Medicine, Gangnam Severance Hospital, Yonsei University College of Medicine, 20, Eonju-ro 63-gil, Gangnam-gu, Seoul, 06229 South Korea; 10 Present Address: Department of Health Promotion, Health Promotion Center, Severance Hospital, Yonsei University Health System, Seoul, South Korea; 11 Present Address: Division of Cardiology, Hallym University Sacred Heart Hospital, Anyang, South Korea

**Keywords:** Peripheral artery disease, Endovascular treatment, Diabetes mellitus, Glycated hemoglobin A, Glucose control, Clinical outcomes

## Abstract

**Background:**

The influence of intensive glucose control in diabetic patients on the macrovascular outcomes is controversial. Thus, this study aimed to elucidate the effect of preprocedural hemoglobin A1c (HbA1c) on clinical outcomes after endovascular therapy for lower extremity artery disease (LEAD) in diabetic patients.

**Methods:**

Diabetic patients were enrolled from the retrospective cohorts of a Korean multicenter endovascular therapy registry and were divided according to the HbA1c level during index admission into the optimal (< 7.0%) or suboptimal (≥ 7.0%) glycemic control groups. The primary endpoints were major adverse limb events (MALE, a composite of major amputation, minor amputation, and reintervention).

**Results:**

Of the 1103 patients enrolled (897 men, mean age 68.2 ± 8.9 years), 432 (39.2%) were classified into the optimal glycemic control group and 671 (60.8%) into the suboptimal glycemic control group. In-hospital events and immediate procedural complications were not different between the two groups. The suboptimal group showed a trend towards a higher incidence of MALE than the optimal group (log-rank p = 0.072). Although no significant differences were found between the two groups in terms of overall survival or amputation, the risk of reintervention was significantly higher in the suboptimal group (log-rank p = 0.048). In the multivariate Cox regression model, suboptimal glycemic control was one of the independent predictors for reintervention. When our data were analyzed according to the initial presentation, suboptimal preprocedural HbA1c significantly increased the incidence of MALE compared with optimal preprocedural HbA1c only in patients with intermittent claudication.

**Conclusion:**

In diabetic patients undergoing endovascular therapy for LEAD, suboptimal preprocedural HbA1c is associated with an increased risk of adverse limb events, especially in patients with intermittent claudication. Further prospective research will be required to validate the role of more intensive glycemic control on the reduction of adverse limb events in diabetic patients undergoing endovascular therapy for LEAD.

## Background

Diabetes mellitus (DM) is associated with the development of peripheral artery disease (PAD) [1]. Both the duration and severity of DM are associated with increased risk of PAD [2, 3]. The prognosis of PAD is worse in DM patients than in non-DM patients. Infrapopliteal arterial involvement is more common and the need for a major amputation is higher in DM patients than in non-DM patients [4, 5].

The cardiovascular effect of intensive glucose control on the macrovascular events among DM patients is controversial. In randomized trials, intensive glucose control in DM patients did not reduced major cardiovascular events compared with standard glucose management [6–8]. However, several studies suggested that poor glycemic control at the time of peripheral angioplasty was associated with worse clinical outcomes in patients with critical limb ischemia (CLI) [9, 10]. In a recent retrospective analysis of US veterans undergoing lower extremity revascularization, patients with lower extremity artery disease (LEAD) and poor glycemic control were at higher risk of amputation and modified major adverse limb events than were those with good glycemic control [11].

The present study aimed to investigate the effect of suboptimal preprocedural hemoglobin A1c (HbA1c) on clinical outcomes of endovascular therapy in LEAD patients with DM using a nationwide, multicenter, real-world registry.

## Methods

### Study population

The Korean Vascular Intervention Society Endovascular therapy in Lower Limb Artery diseases (K-VIS ELLA) registry is a multicenter observational study with retrospective and prospective cohorts of patients with lower extremity artery disease treated with endovascular therapy (ClinicalTrials.gov NCT02748226). The retrospective patient cohort consisted of 3434 patients with 5097 affected limbs treated between January 2006 and July 2015 in 31 Korean hospitals. The K-VIS ELLA registry study design and results have been described in detail previously [12]. A total of 3073 patients with 3972 target limbs were finally analyzed after exclusion of 56 limbs with acute limb ischemia, 82 limbs with Buerger’s disease, 11 limbs lacking procedural or in-hospital data, 528 limbs lacking follow-up data after hospital discharge, and 448 limbs treated for repeat revascularization following the index procedure.

**Fig. 1:**
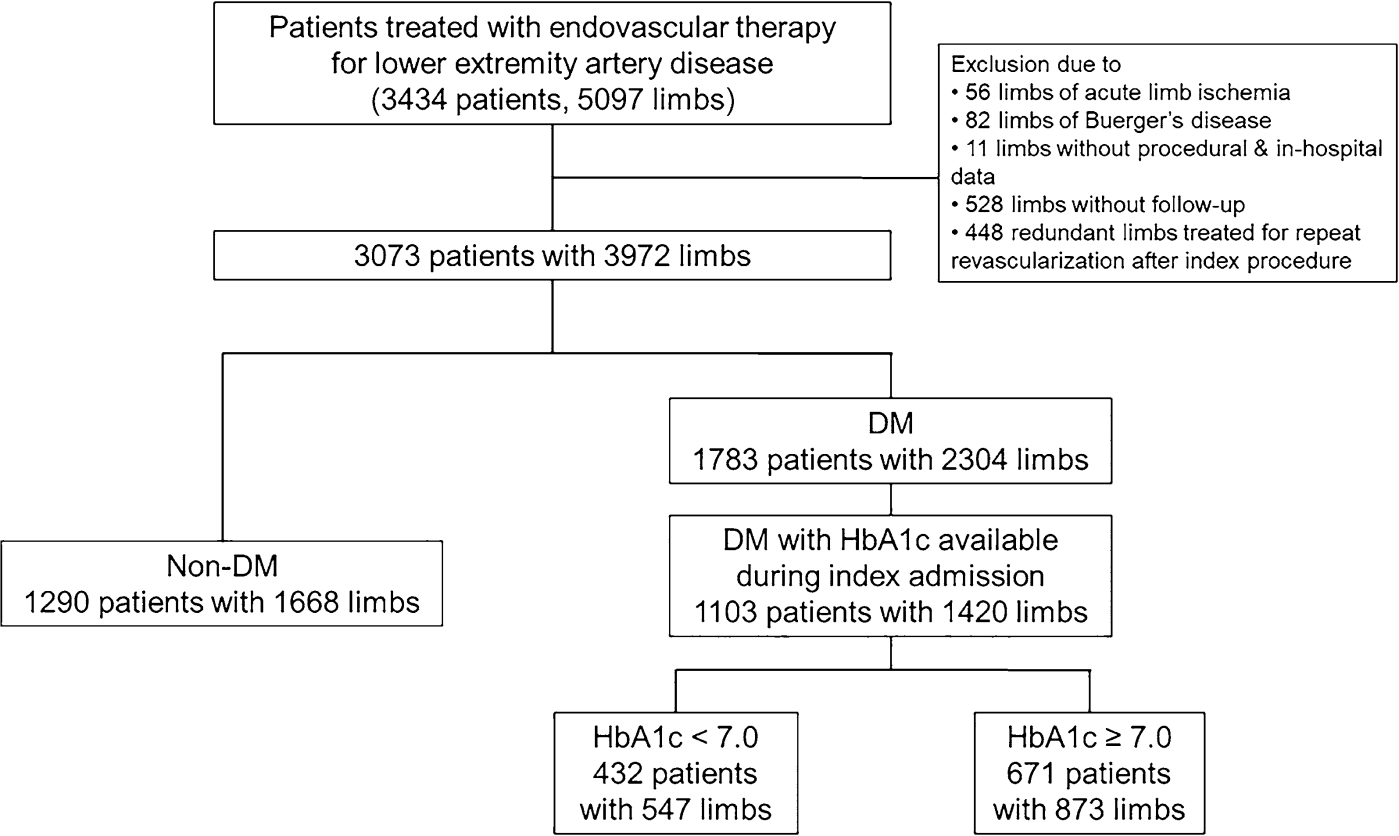
Study population flow chart. *DM* diabetes mellitus, *HbA1c* hemoglobin A1c

From this registry population, 1103 DM patients (1420 limbs) who had HbA1c levels available during index admission were finally included in the current analysis (Fig. 1). HbA1c levels were dichotomized into optimal (< 7.0%) and suboptimal (≥ 7.0%) according to American Diabetes Association recommendations [13]. Data on patient demographics, baseline clinical and lesion characteristics, medication history, clinical presentation, laboratory test results, treatments, and follow-up outcomes were collected from electronic medical records.

The study protocol was approved by the institutional review board of each hospital and was conducted according to the principles of the Declaration of Helsinki. The institutional review boards of the participating hospitals waived the requirement of informed consent due to the retrospective nature of the study.

### Definitions and study endpoints

LEAD was defined as the presence of ≥ 50% narrowing of a lower extremity artery. Claudication was defined as Rutherford category 1, 2, or 3 disease and CLI was defined as Rutherford category 4, 5, or 6 disease [14]. The presence of diabetes was identified by history and medical records including outpatient clinics and prescriptions of oral hypoglycemic agents or insulin. Definitions of hypercholesterolemia, smoking, congestive heart failure, anemia, and chronic kidney disease were described in a previous report [12].

Technical success was defined as successful revascularization with residual stenosis < 30% and absence of flow-limiting dissection or a hemodynamically significant translesion pressure gradient. Major amputation was defined as any lower extremity amputation at the level of or above the ankle, and a minor amputation was defined as any lower extremity amputation below the ankle, including the foot or toe(s).

The primary endpoints of this study were major adverse limb events (MALE; a composite of major amputation, minor amputation, and reintervention). Secondary endpoints were all-cause mortality, any amputation, and reintervention. These outcomes were compared between optimal glycemic control and suboptimal glycemic control groups according to the HbA1c level during the index admission.

### Statistical analysis

Continuous variables were expressed as mean ± standard deviation and were compared using the Student’s t-test for parametric data and the Mann–Whitney test for nonparametric data. Categorical variables were expressed as number (percentage) and were compared using the Chi square test or Fisher’s exact test. Data were analyzed on a per-patient basis for clinical characteristics and on a per-lesion basis for the limb, lesion, or procedural characteristics. Cumulative incidences of clinical events were presented as Kaplan–Meier estimates and were compared using the log-rank test. Univariate Cox proportional hazards regression analyses using baseline clinical, lesion, and procedural variables were performed to identify factors associated with clinical events. The variables achieving P-values < 0.20 in the univariate analysis were evaluated in the multivariate analysis model to determine the independent predictors of clinical events. All statistical analyses were performed using SPSS (version 23.0; IBM Corp., Armonk, NY, USA). All tests were two-sided, and P < 0.05 was considered statistically significant.

## Results

### Baseline characteristics

Baseline clinical characteristics of patients according to HbA1c levels during index admission are summarized in Table 1. Of the 1103 patients enrolled, 432 (39.2%) were categorized into the optimal glycemic control group (HbA1c < 7.0) and 671 (60.8%) were placed into the suboptimal glycemic control group (HbA1c ≥ 7.0). The mean age of the entire cohort was 68.2 ± 8.9 years, and 897 (81.3%) patients were men. Compared with the patients in the suboptimal group, those in the optimal group were older, were predominantly male, and had a higher prevalence of chronic kidney disease and congestive heart failure. Patients in the suboptimal group were more likely to be on insulin therapy for glycemic control. Initial clinical presentations based on the Rutherford classification were not different between the two groups.

**Table 1:** Baseline clinical characteristics

	Total (n = 1103)	HbA1c < 7.0 (n = 432)	HbA1c ≥ 7.0 (n = 671)	p
Age (years)	68.2 ± 8.9	69.4 ± 8.9	67.5 ± 8.7	0.001
Old age (≥ 80 years)	96 (8.7%)	48 (11.1%)	48 (7.2%)	0.028
Male	897 (81.3%)	365 (84.5%)	532 (79.3%)	0.033
Hemoglobin A1c (%)	7.6 ± 1.6	6.3 ± 0.5	8.5 ± 1.4	< 0.001
BMI (kg/m^2^)	24.1 ± 3.8	24.0 ± 3.9	24.2 ± 3.7	0.607
Low BMI (< 18.5 kg/m^2^)	46 (4.4%)	19 (4.7%)	27 (4.2%)	0.757
Hypertension	870 (78.9%)	347 (80.3%)	523 (77.9%)	0.365
Use of Insulin	361 (32.7%)	109 (25.2%)	252 (37.6%)	< 0.001
Hypercholesterolemia	426 (38.6%)	176 (40.7%)	250 (37.3%)	0.255
Current smoker	288 (26.1%)	101 (23.4%)	187 (27.9%)	0.106
Chronic kidney disease	279 (25.3%)	124 (28.7%)	155 (23.1%)	0.040
End-stage renal disease	166 (15.0%)	72 (16.7%)	94 (14.0%)	0.262
Coronary artery disease	653 (59.2%)	263 (60.9%)	390 (58.1%)	0.380
Congestive heart failure	57 (5.2%)	30 (6.9%)	27 (4.0%)	0.037
Previous history of stroke	185 (16.8%)	81 (18.8%)	104 (15.5%)	0.161
Previous history of EVT	99 (9.0%)	46 (10.6%)	53 (7.9%)	0.131
Previous history of bypass surgery	28 (2.5%)	10 (2.3%)	18 (2.7%)	0.845
Previous history of amputation	81 (7.3%)	33 (7.6%)	48 (7.2%)	0.813
Rutherford classification				0.201
1	89 (8.1%)	40 (9.3%)	49 (7.3%)	
2	259 (23.5%)	106 (24.5%)	153 (22.8%)	
3	272 (24.7%)	106 (24.5%)	166 (24.7%)	
4	80 (7.3%)	33 (7.6%)	47 (7.0%)	
5	236 (21.4%)	80 (18.5%)	156 (23.2%)	
6	167 (15.1%)	67 (15.5%)	100 (14.9%)	

Values are presented as mean ± standard deviation or number (%) unless otherwise stated

*BMI* body mass index, *EVT* endovascular therapy

### Procedural characteristics and complications

**Fig. 2:**
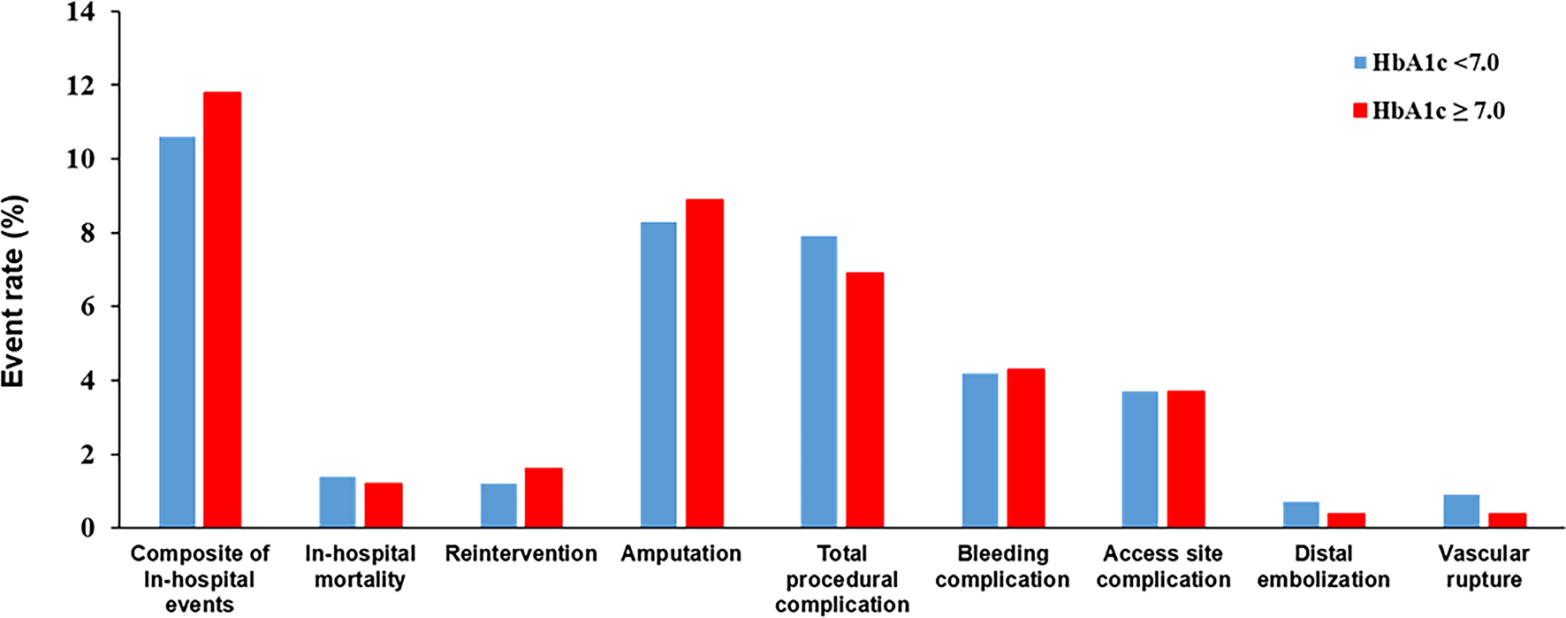
In-hospital outcomes and procedural complication rates according to HbA1c level during index admission. Crude incidence of death, reintervention, amputation, bleeding complication, access site complication, distal embolization, and vascular rupture for optimal (blue) and suboptimal (red) glucose control groups. *HbA1c* hemoglobin A1c

Table 2 demonstrates baseline lesion and procedural characteristics according to HbA1c levels during the index admission. No significant differences were found between the groups except that there were slightly more targeted blood vessels in the suboptimal group. Total in-hospital events and immediate procedural complications were not different between the two groups (Fig. 2).

**Table 2:** Baseline lesion and procedural characteristics

	Total (n = 1420 limbs)	HbA1c < 7.0 (n = 547 limbs)	HbA1c ≥ 7.0 (n = 873 limbs)	p
Ankle brachial index	0.7 ± 0.3	0.7 ± 0.3	0.7 ± 0.3	0.777
TASC II classification				0.543
A	224 (15.8%)	95 (17.4%)	129 (14.8%)	
B	310 (21.8%)	122 (22.3%)	188 (21.5%)	
C	304 (21.4%)	114 (20.8%)	190 (21.8%)	
D	582 (41.0%)	216 (39.5%)	366 (41.9%)	
Number of target vessels	1.5 ± 0.7	1.4 ± 0.6	1.5 ± 0.8	0.006
Target vessels				0.058
Aortoiliac	461 (32.5%)	192 (35.1%)	269 (30.8%)	
Femoropopliteal	666 (46.9%)	253 (46.3%)	413 (47.3%)	
Infrapopliteal	293 (20.6%)	102 (18.6%)	191 (21.9%)	
Total occlusion	592 (41.7%)	235 (43.0%)	357 (40.9%)	0.472
In-stent restenosis	45 (3.2%)	20 (3.7%)	25 (2.9%)	0.438
Treatment modality				0.918
Balloon only	582 (41.0%)	219 (40.0%)	363 (41.6%)	
Stent	810 (57.0%)	321 (58.7%)	489 (56.0%)	
Others	28 (2.0%)	7 (1.3%)	21 (2.4%)	
Lesion length (mm)	111.7 ± 97.6	111.1 ± 96.1	112.1 ± 98.6	0.856
Diameter stenosis (%)	88.0 ± 14.1	88.4 ± 14.4	87.9 ± 14.0	0.553

Values are presented as mean ± standard deviation or number (%) unless otherwise stated

*TASC II* Trans-Atlantic Inter-Society Consensus Document on Management of Peripheral Arterial Disease II Classifications

### Follow-up clinical outcomes and independent predictors

**Fig. 3:**
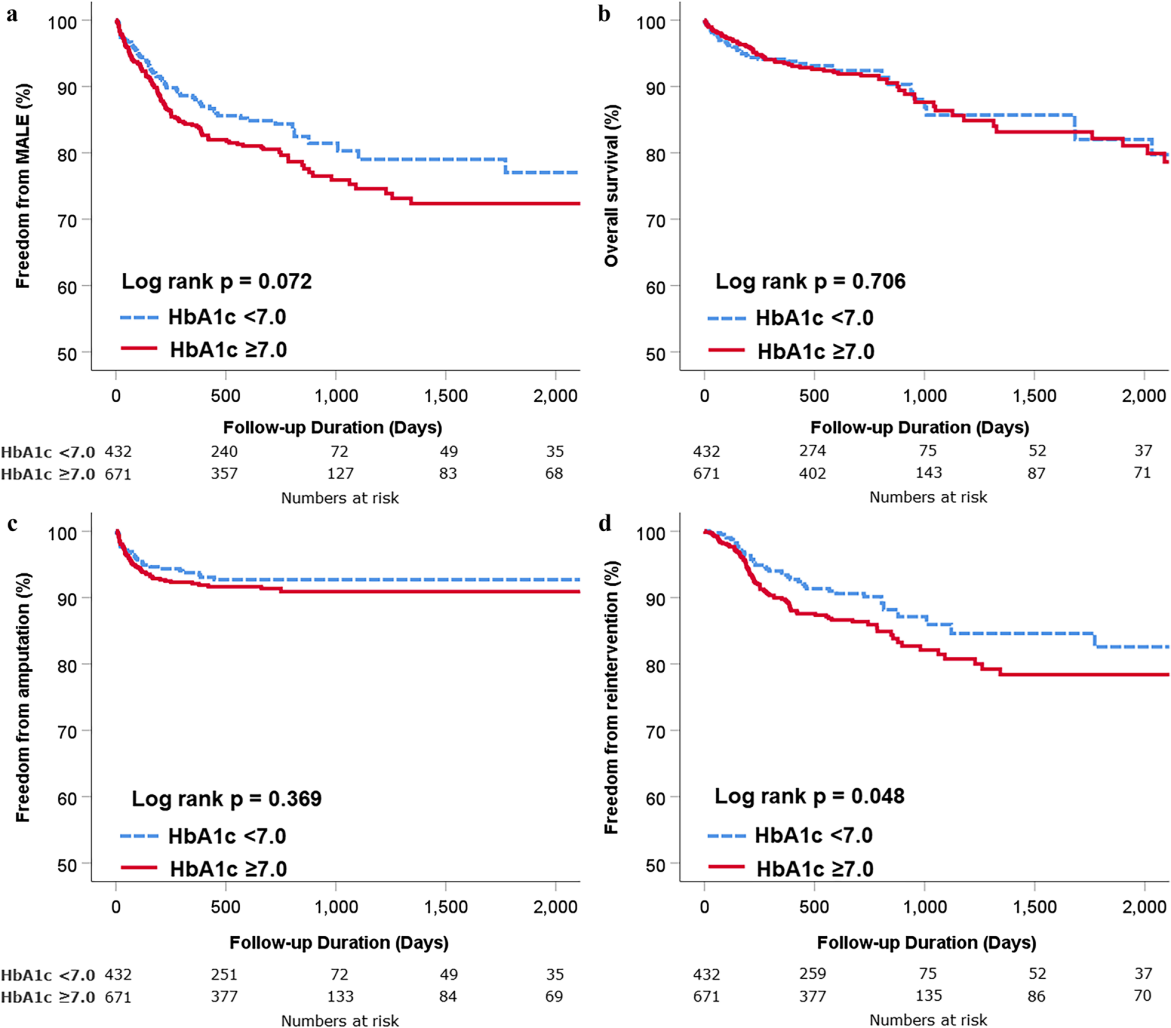
Comparison of clinical outcomes according to HbA1c level during index admission. Kaplan–Meier curves for comparison of freedom from MALE (**a**), death (**b**), amputation (**c**), and reintervention (**d**). *HbA1c* hemoglobin A1c, *MALE* major adverse limb events

The median follow-up duration was 724 days (interquartile range 326–782 days). The Kaplan–Meier curves in Fig. 3 illustrate freedom from MALE, death, amputation, and reintervention stratified by initial HbA1c status. The suboptimal group showed a trend toward higher incidence of MALE than the optimal group (log-rank p = 0.072, Fig. 3a). No significant differences were observed between the two groups in terms of overall survival (Fig. 3b) or amputation (Fig. 3c) during the follow-up period. However, the suboptimal group demonstrated a significant disadvantage with regard to reintervention (log-rank p = 0.048, Fig. 3d). In the multivariate Cox regression model, the use of insulin for glycemic control, end-stage renal disease, previous history of amputation, previous history of endovascular therapy, and CLI were independent predictors of MALE (Table 3). Independent predictors for reintervention during follow-up were end-stage renal disease, previous history of endovascular therapy, and suboptimal glycemic control defined as HbA1c ≥ 7.0% (Table 4).

**Table 3:** Independent predictors of major adverse limb events

	Univariable analysis			Multivariable analysis		
	HR	95% CI	p-value	HR	95% CI	p-value
Age (per 10 years)	0.824	0.702–0.967	0.018	0.887	0.748–1.051	0.167
Sex (female)	1.200	0.847–1.700	0.306			
Low BMI (< 18.5 kg/m^2^)	1.732	0.965–3.109	0.066	0.593	0.329–1.071	0.083
Hypertension	0.815	0.587–1.130	0.219			
Use of insulin	1.944	1.462–2.585	< 0.001	1.386	1.020–1.885	0.037
Hypercholesterolemia	0.836	0.622–1.123	0.234			
End-stage renal disease	2.727	1.960–3.794	< 0.001	1.776	1.238–2.546	0.002
Congestive heart failure	1.622	0.957–2.749	0.073	1.563	0.903–2.705	0.111
Current smoker	0.658	0.464–0.933	0.019	0.711	0.495–1.022	0.065
Coronary artery disease	1.131	0.784–1.632	0.509			
Previous history of stroke	0.743	0.414–1.333	0.319			
Previous history of Bypass surgery	1.150	0.473–2.796	0.758			
Previous history of amputation	2.526	1.681–3.796	< 0.001	1.564	1.016–2.408	0.042
Previous history of EVT	1.667	1.101–2.526	0.016	1.858	1.210–2.853	0.005
Critical limb ischemia	2.472	1.845–3.312	< 0.001	1.970	1.426–2.723	< 0.001
HbA1c ≥ 7.0%	1.319	0.975–1.786	0.073	1.269	0.927–1.738	0.137

*BMI* body mass index, *CI* confidence interval, *EVT* endovascular therapy, *HbA1c* hemoglobin A1c, *HR* hazard ratio

**Table 4:** Independent predictors of reintervention

	Univariable analysis			Multivariable analysis		
	HR	95% CI	P-value	HR	95% CI	P-value
Age (per 10 years)	0.963	0.790–1.174	0.709			
Sex (female)	1.018	0.816–1.271	0.872			
Low BMI (< 18.5 kg/m^2^)	1.445	0.674–3.098	0.344			
Hypertension	0.740	0.502–1.091	0.128	0.767	0.516–1.140	0.189
Use of insulin	1.394	0.978–1.987	0.066	1.147	0.789–1.668	0.472
Hypercholesterolemia	1.182	0.834–1.675	0.346			
End-stage renal disease	1.725	1.095–2.718	0.019	1.719	1.074–2.753	0.024
Congestive heart failure	1.225	0.598–2.506	0.579			
Current smoker	0.899	0.607–1.330	0.593			
Coronary artery disease	0.699	0.495–0.987	0.042	0.863	0.584–1.274	0.458
Previous history of stroke	1.023	0.646–1.618	0.924			
Previous history of bypass surgery	1.846	0.754–4.518	0.180	1.598	0.637–4.009	0.318
Previous history of amputation	1.547	0.872–2.745	0.136	1.172	0.649–2.118	0.598
Previous history of EVT	2.230	1.406–3.537	0.001	2.333	1.455–3.740	< 0.001
Critical limb ischemia	1.194	0.839–1.700	0.325			
HbA1c ≥ 7.0%	1.458	1.001–2.123	0.049	1.473	1.005–2.160	0.047

*BMI* body mass index, *CI* confidence interval, *EVT* endovascular therapy, *HbA1c* hemoglobin A1c, *HR* hazard ratio

### Influence of preprocedural HbA1c levels on clinical outcomes according to the initial presentation

**Fig. 4:**
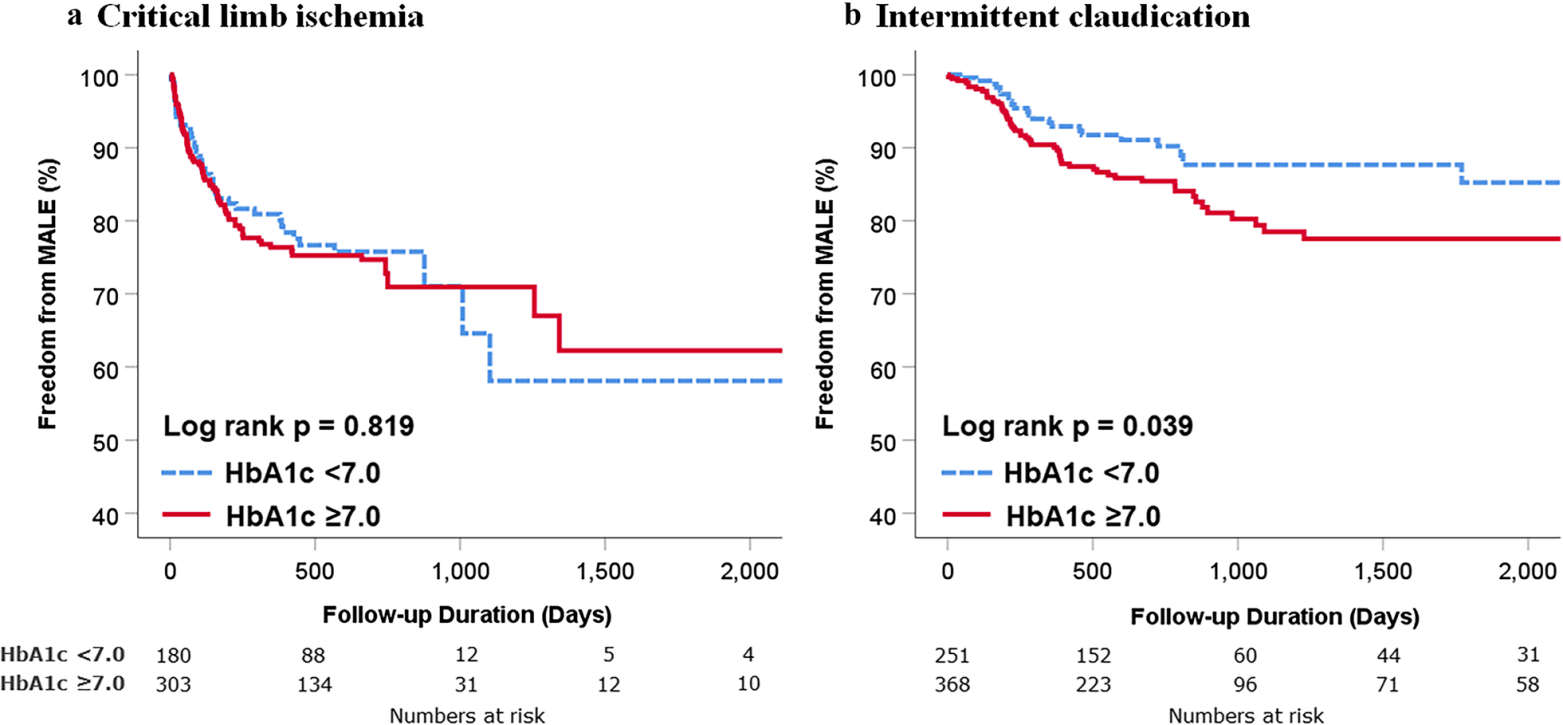
Comparison of MALE according to HbA1c level during index admission. Kaplan–Meier curves for comparison of freedom from MALE in patients with critical limb ischemia (**a**) or intermittent claudication (**b**). *HbA1c* hemoglobin A1c, *MALE* major adverse limb events

To assess the influence of preprocedural HbA1c levels on clinical outcomes according to the initial clinical presentation, we analyzed data separately in patients with intermittent claudication or CLI (Fig. 4). Among CLI patients, no difference was found in the incidence of MALE between the optimal and suboptimal groups (Fig. 4a). However, among patients who presented with claudication, the suboptimal group showed a higher incidence of MALE than the optimal group (log rank p = 0.039, Fig. 4b).

## Discussion

### Main findings

We reported the association between the preprocedural glycemic control based on HbA1c during index admission and clinical outcomes in DM patients undergoing endovascular therapy for LEAD. Although not statistically significant, the suboptimal glycemic control group (HbA1c ≥ 7.0) showed a tendency towards higher incidence of MALE than in the optimal glycemic control group (HbA1c < 7.0). Elevated preprocedural HbA1c was associated with higher risk of reintervention. Suboptimal glycemic control was an independent predictor of reintervention during the follow-up period after endovascular therapy in DM patients with LEAD. Because CLI was one of the independent predictors for MALE, we further analyzed our data by dividing it into the presence of claudication and CLI according to the initial presentation. Suboptimal preprocedural HbA1c did not affect the outcome in CLI patients; however, among those with intermittent claudication, suboptimal preprocedural HbA1c significantly increased the incidence of MALE compared with optimal glycemic control.

### Role of glycemic control on vascular outcomes

The role of intensive glucose control on the macrovascular outcomes in DM patients is unclear. Although large randomized controlled trials failed to demonstrate the benefits of intensive glucose control in reducing major cardiovascular events [6–8], recent post hoc analyses of several randomized trials demonstrated that higher HbA1c was associated with increased risk of major cardiovascular events [15, 16] and lower extremity amputation [17].

For the management of LEAD, recent practice guidelines recommended adequate glycemic control in DM patients [18–20], and several retrospective observational studies suggested that poor glycemic control may be associated with worse clinical outcomes of vascular procedure in DM patients with LEAD. In a single-center study of 278 CLI patients undergoing balloon angioplasty, Takahara et al. reported that the presence of diabetes was independently associated with major amputation and, in the DM subgroup, poor glycemic control was associated with higher amputation rates [9]. Similarly, in another single-center study, Singh et al. analyzed the effect of pre-procedural fasting blood glucose on primary patency and limb outcomes following infrapopliteal balloon angioplasty in 149 DM patients with CLI [10]. They found that fasting blood glucose above the median value at the time of the procedure was associated with lower primary patency at 1 year [10]. However, these were single-center studies with relatively small sample size and only CLI patients were enrolled. Therefore, the results cannot be generalized to all LEAD patients.

Recently, Arya et al. reported a large retrospective study using Veterans Health Administration data to evaluate the influence of elevated perioperative HbA1c on outcomes in LEAD patients undergoing surgical or endovascular revascularization procedures [11]. In that study, high HbA1c level was incrementally associated with higher risk of amputation and MALE regardless of the preoperative diagnosis of DM [11]. In the present study, we found no significant differences between the two groups in terms of risk of amputation or MALE, although the suboptimal group showed a trend toward higher incidence of MALE than the optimal group. Compared with the aforementioned studies, the relatively lower incidence of amputation in our study groups may be one of the reasons for the differences. Furthermore, the subjects of Arya et al.’s study were veterans, most of whom were male, and approximately 40% of the subjects were not diagnosed with DM preoperatively. Furthermore, LEAD severity was not specified in more than a third of the enrolled patients. These might be possible reasons for the discrepancies between our results and those of Arya et al. Recent studies in patients undergoing open vascular surgery demonstrated that poor glycemic control was associated with earlier postoperative outcomes such as in-hospital limb events [21] or 30-day mortality [22, 23]. Compared with the results from studies in patients undergoing endovascular therapy, earlier effect of poor glycemic control on postoperative outcomes observed in patients undergoing vascular surgery may be attributed to surgery-related complications such as wound complications or postoperative infections.

When our data were analyzed separately in two groups according to initial presentation, the benefit of optimal glycemic control in reducing the risk of MALE was observed only in patients presenting with claudication. Considering the seriousness of the CLI as an independent risk factor for MALE, intensive glucose control possibly did not significantly reduce the incidence of MALE in CLI patients. Relatively lower incidence of amputation in our study may also have affected the results. A recent study evaluated the association between preoperative HbA1c levels and clinical outcomes after lower extremity bypass surgery and found that poor preoperative glycemic control increased the risk of in-hospital limb events [21]. Similar to our findings, the increased risk for adverse limb events in patients with high preoperative HbA1c level was only observed in patients without CLI [21].

### Other factors related to PAD outcomes

In addition to HbA1c, various biomarkers have been suggested to be related to clinical outcomes of PAD in DM patients. Zhao et al. investigated the association of vascular damage with the triglyceride-glucose index as a simple surrogate marker of insulin resistance [24]. They demonstrated that an increased triglyceride-glucose index was significantly associated with a higher risk of arterial stiffness and renal microvascular damage, but not with PAD [24]. Biscetti et al. demonstrated that circulating sortilin levels were associated with the presence and severity of LEAD in statin-naïve DM patients [25]. Biscetti et al. found that omentin-1 serum levels were significantly lower in DM patients with PAD than in DM patients without PAD, and omentin-1 levels were related to disease severity [26]. Elevated levels of various inflammatory cytokines such as osteoprotegerin, tumor necrosis factor-a, interleukin-6, and C-reactive protein were also reported to be related with worse vascular outcomes in DM patients with LEAD undergoing infrapopliteal endovascular therapy [27]. These potential surrogate biomarkers may be helpful to better stratify PAD risk in DM patients. Their usefulness should be confirmed in future studies.

In addition to molecular biomarkers, several clinical confounding factors may influence on clinical outcomes of LEAD in DM patients. It has been shown that women with LEAD have more functional impairment and worse outcomes than men [28]. Although these gender differences were not observed in the present study population, in a previous study performed on the entire cohort from our registry, women had higher rates of major adverse cardiovascular events and MALE [29]. Frailty is also associated with poor clinical outcomes after vascular interventions for LEAD [30], although increasing numbers of peripheral vascular intervention for older patients was shown to correlate with decreasing major amputation rates [31].

## Limitations

This study has several limitations. First, given the retrospective nature of the study, the causal relationships could not be determined. Therefore, prospective randomized trials will be required to validate the role of more intensive glycemic control on the reduction of adverse limb events in DM patients with LEAD undergoing endovascular therapy. Second, the HbA1c level that we used as the basis of glycemic control was measured once before the procedure, and data on changes in glycemic control during the follow-up period were not available. Therefore, dramatic changes in blood glucose levels during follow-up may have affected the clinical outcomes. However, HbA1c reflects the average blood glycemic level over approximately 3 months [13], and the measurement of HbA1c was performed during the index admission in all cases. At least our data highlight the importance of glycemic control during preprocedural period. Third, the strategy for hyperglycemia management was not standardized. Recent studies have shown that novel glucose-lowering agents can reduce the risk of cardiovascular events in patients with atherosclerotic cardiovascular disease [32]. However, increased risk of lower limb amputation has been reported for certain sodium-glucose cotransporter type-2 inhibitors in patients with LEAD [33]. For these reasons, the optimal method of glycemic control is an important topic to be addressed in future studies.

## Conclusion

In DM patients undergoing endovascular therapy for LEAD, suboptimal preprocedural HbA1c is associated with increased risk of adverse limb events, especially in patients with intermittent claudication. Future research will have to determine whether more intensive glycemic control can improve clinical outcomes after endovascular therapy in DM patients with LEAD.

## Abbreviations

CLI: Critical limb ischemia
DM: Diabetes mellitus
HbA1c: Hemoglobin A1c
LEAD: Lower extremity artery disease
MALE: Major adverse limb events
PAD: Peripheral artery disease
